# Eating disorders and health literacy in Germany: results from two representative samples of adolescents and adults

**DOI:** 10.3389/fpsyg.2024.1464651

**Published:** 2024-09-16

**Authors:** Lars König, Rebekka Schröder, Tim Hamer, Ralf Suhr

**Affiliations:** ^1^Stiftung Gesundheitswissen, Berlin, Germany; ^2^Institut für Medizinische Soziologie und Rehabilitationswissenschaft, Charité – Universitätsmedizin Berlin, Berlin, Germany

**Keywords:** eating disorder, health literacy, body image, health, representative, Germany, adolescents, adults

## Abstract

**Introduction:**

Eating disorders are associated with substantial burden for the affected individuals including negative health outcomes and increased mortality. So far, prevention programs for eating disorders have yielded mixed results concerning their efficacy. Therefore, more targeted prevention programs need to be developed. Health literacy has been identified as a potential influencing factor of eating disorders. This study aimed at exploring the relationship between likely cases of eating disorders and health literacy, alongside additional sociodemographic factors.

**Materials and methods:**

Two large samples of adults (*N* = 3,011) and adolescents (*N* = 1,021) representative of the German-speaking population in Germany were recruited. Likely cases of eating disorders were identified using the SCOFF questionnaire. Health literacy was assessed with the HLS-EU-Q16 questionnaire. Sociodemographic information, including age, gender, social status and level of education, and subjective body image were obtained. χ^2^-tests of independence were calculated to determine the association between the investigated constructs.

**Results:**

Suspected eating disorders were more likely in female than male adolescents but were not related to gender in adults. Rates of suspected eating disorders increased with increasing age in adolescents and decreased with increasing age in adults. While levels of education were unrelated to suspected eating disorders, low social status was associated with higher rates of suspected eating disorders in adults but not adolescents. Inadequate or problematic health literacy and negative body image were associated with higher rates of suspected eating disorders compared to adequate health literacy and more positive body image.

**Discussion:**

Likely cases of eating disorders are related to health literacy and body image as well as sociodemographic factors. These constructs should therefore be addressed in future research to improve prevention programs.

## 1 Introduction

Eating disorders, such as anorexia nervosa, bulimia nervosa and binge-eating disorder, are severe psychiatric disorders associated with significant psychological and physical impairment (Treasure et al., 2020; World Health Organization, 2019). Eating disorders are characterized by abnormal eating or weight-control behaviors often accompanied by disturbed body image (Treasure et al., 2020). Patients with anorexia nervosa show a persistent pattern of restrictive eating or other behaviors that are aimed at establishing or maintaining abnormally low body weight (World Health Organization, 2019). Both bulimia nervosa and binge-eating disorder are characterized by recurrent binge-eating episodes, in which individuals experience loss of control over their eating behavior and consume large portions of food in a discrete period of time (Treasure et al., 2020; Giel et al., 2022; World Health Organization, 2019). For patients with bulimia nervosa, these episodes are accompanied by compensatory behaviors such as self-induced vomiting to prevent weight gain (Treasure et al., 2020), which do not regularly occur in binge-eating disorder (Giel et al., 2022).

On a global level, 12-months prevalences of all eating disorders are estimated at 2.2% for women and 0.7% for men (Galmiche et al., 2019). For a majority of patients with eating disorders, age of eating disorder onset is before 25 years with a peak age of 15 years (Preti et al., 2009; Solmi et al., 2022). Results concerning a potential increase in eating disorder prevalences in recent years are heterogeneous (Keski-Rahkonen and Mustelin, 2016; Steinhausen and Jensen, 2015). However, a rise in hospital admissions in patients with eating disorders, especially in anorexia nervosa, has been observed, which was further accelerated during the COVID-19 pandemic in recent years (Devoe et al., 2023; Holland et al., 2016).

Although eating disorder prevalences are still relatively low compared to other psychiatric conditions, eating disorders have serious implications, both at an individual and societal level (Chan et al., 2023; Chesney et al., 2014; GBD 2019 Mental Disorders Collaborators, 2022; Ágh et al., 2016). Specifically, eating disorders are associated with high rates of psychiatric comorbidities such as mood and anxiety disorders, physical conditions including cardiovascular and metabolic problems due to malnutrition, weight loss and compensatory behaviors as well as decreased quality of life (Keski-Rahkonen and Mustelin, 2016; Preti et al., 2009; Treasure et al., 2020; Westmoreland et al., 2016; Ágh et al., 2016). When compared to other mental disorders, eating disorders—after substance-abuse disorders—have the second highest number of years-of-potential-life-lost (Chan et al., 2023). Furthermore, eating disorders, particularly anorexia nervosa, are among the psychiatric disorders with the highest mortality rates (Harris and Barraclough, 1998; Arcelus et al., 2011), as evidenced by standardized mortality ratios five to six times that of the general population (Arcelus et al., 2011). Also, while many patients with eating disorders eventually recover, chronicity of the disorders occurs in almost a quarter of patients (Solmi et al., 2024).

The etiology of eating disorders is still subject of ongoing debate. In addition to biological risk factors (e.g., genetics, microbiome), environmental and psychological risk factors play a crucial role in developing eating disorders. These include sexual and physical abuse, trauma and childhood obesity, higher educational attainment, body image-related factors and use of appearance-focused social media, among others (Barakat et al., 2023; Solmi et al., 2021).

Although there are effective treatments available for all major eating disorders (Herpertz et al., 2018; Monteleone et al., 2022; Murray et al., 2019) not all individuals with eating disorders seek or receive treatment (Hart et al., 2011). Among the common barriers that prevent individuals from getting treatment are stigma and shame about the disorder, denial or failure to recognize the severity of symptoms, and practical barriers such as the cost and accessibility of treatments (Ali et al., 2017).

In a recent meta-analysis of a widely-used screening inventory that is also employed in the present study, 22.4% of children and adolescents (age 7–18 years) were identified as suspected of having an eating disorder, with rates in girls (30.0%) almost double those in boys (17.0%) (López-Gil et al., 2023). Similar or slightly lower rates of potential eating disorders were also found in three German-speaking samples (Herpertz-Dahlmann et al., 2008; Cohrdes et al., 2019; Zeiler et al., 2016). Critically, positive screens for eating disorders in younger age are not only predictive of potential underlying full-blown eating disorders but also of current quality of life (Zeiler et al., 2016), as well as overweight, obesity and depressive symptoms later in life (Herpertz-Dahlmann et al., 2015).

The numerous risk factors for eating disorders (Barakat et al., 2023; Solmi et al., 2021) and the substantial burden of the diseases (Ágh et al., 2016; Keski-Rahkonen and Mustelin, 2016; GBD 2019 Mental Disorders Collaborators, 2022) underline the need of preventive interventions (Herpertz-Dahlmann et al., 2015). Onset of most eating disorders in adolescence and the fact that recovery rates are higher in adolescents than in adults and in patients at earlier vs. later stages of the disorder suggest that targeted interventions for prevention or early detection and intervention in children and adolescents may prove to be particularly valuable (Steinhausen and Jensen, 2015; Ambwani et al., 2020; Preti et al., 2009; Kalindjian et al., 2022).

So far, a number of prevention programs to battle eating disorders have been developed with mixed results concerning their efficacy (Koreshe et al., 2023; Le et al., 2017). However, some interventions (e.g., media literacy interventions for universal prevention) have been identified as promising in terms of symptom reductions (Le et al., 2017) and certain intervention programs (e.g., dissonance-based prevention programs) were found to even prevent future onset of eating disorders (Stice et al., 2021). Therefore, identifying groups that might particularly benefit from prevention programs, is vital to improve the efficacy of these programs (Le et al., 2017). The present study was designed to address this need and to explore potential associations with health literacy.

Health literacy refers to the cognitive and social skills which determine the motivation and ability of individuals to gain access to, understand and use information in ways which promote and maintain good health (Nutbeam, 1998). It has been associated with several health-related topics, including disordered eating (Fleary et al., 2018; Campanino et al., 2023; Boberová and Husárová, 2021; Bullivant et al., 2020). For example, it has been shown that individuals with anorexia nervosa and bulimia nervosa had lower levels of subjective health literacy and higher levels of objective health literacy when compared to healthy controls (Campanino et al., 2023). In addition, a negative relationship between health literacy and symptoms of eating disorders was found, particularly in participants who perceived themselves to be overweight (Boberová and Husárová, 2021). Therefore, targeting health literacy in prevention programs could be particularly promising.

The aim of the present investigation was to explore the association between suspected cases of eating disorders and sociodemographic variables, body image and health literacy in two representative samples of German-speaking adolescents (12–17 years) and adults (≥ 16 years) in Germany.

## 2 Materials and methods

### 2.1 Ethical considerations

The study was designed to comply with the ethical principles for medical research involving human subjects as set out in the Declaration of Helsinki. A study protocol was submitted to the ethics committee of the Berlin Medical Association. The ethics committee had no ethical or professional objections to the study protocol (reference Eth-64/23). Before the study started, participants gave their informed consent to take part in the study. For participants aged 15 and younger, informed consent was provided by their parents. Participants could withdraw from the study at any time. Participants were not compensated for their participation by the independent non-profit foundation Stiftung Gesundheitswissen. Furthermore, only anonymized data was provided to the Stiftung Gesundheitswissen.

### 2.2 Survey methodology and data acquisition

For this study, data from two independent samples were collected by two market research institutes.

Data for the adult sample (≥ 16 years) was collected by forsa (Gesellschaft für Sozialforschung und statistische Analysen mbH) in December 2023 using the forsa.omninet online panel. The panel has around 100,000 participants and is representative of the German-speaking population with internet access in Germany. For the present investigation, a representative sample was drawn from the panelists 16 years and older. Data collection was carried out online with computer-assisted web interviews. A total of 3,011 participants provided complete questionnaires. Survey weights were calculated by forsa using an iterative proportional fitting approach according to the following weight variables and combinations: (a) gender × age (in the groups 16–29 years, 30–45 years, 46–64 years, ≥ 65 years) × region (West Germany and Berlin, East Germany) and (b) federal state. The weighting was based on the population census of the German Federal Statistical Office.

Data for the adolescent sample was collected by GIM (Gesellschaft für innovative Marktforschung mBH). The study population consisted of pupils aged 12 to 17 from Germany. Data collection was carried out with a mixed-mode approach (similar to representative German JIM-Studie 2023) using computer-assisted web interviews (about 2/3 of all interviews) and computer-assisted face-to-face interviews (about 1/3 of all interviews). A total of 1,021 complete questionnaires were obtained. Data collection was carried out in November and December 2023. Survey weights were calculated in an iterative procedure according to the following weight variables and combinations: age × gender, education and federal state. The weighting was based on data from the Federal Statistical Office and the ma Radio survey.

### 2.3 Measures

#### 2.3.1 Sociodemographic information

Participants provided basic sociodemographic information including gender (male, female, diverse [in adolescents only]), age and federal state of residence (in the adult sample) or federal state of the currently attended schools (in the adolescent sample). For further analyses, individuals were divided into groups, according to age (16–29 years, 30–45 years, 46–64 years, ≥ 65 years, in the adult sample; 12–13 years, 14–15 years, 16–17 years, in the adolescent sample) and gender (male, female, diverse [in adolescents only]).

In addition, information on (subjective) social status and educational levels were obtained.

Adults were asked to indicate their highest level of formal education. Participants were then categorized into three groups, i.e., low (equivalent to no formal education or basic secondary school; ohne Haupt-/Volksschulabschluss, Haupt-/Volksschulabschluss), middle (equivalent to intermediate secondary school; Mittlere Reife, Realschulabschluss, Fachschulreife, Abschluss der Polytechnischen Oberschule, Fachhochschulreife, Abschluss einer Fachoberschule) and high formal level of education (equivalent to most advanced secondary school, e.g., grammar schools to obtain a general or specialized university entrance qualification, or university degree; Abitur, allgemeine oder fachgebundene Hochschulreife, Fach-/Hochschulstudium). To obtain level of education from adolescents, they were first asked to indicate the type of school they currently attend and then divided into two categories: high (equivalent to most advanced secondary school, e.g., grammar schools, where a general university entrance qualification can be obtained; Gymnasium) and low educational levels (all other school types).

Subjective social status of the adult participants was assessed using the German version of the MacArthur scale (Hoebel et al., 2015; Adler et al., 2000). The scale makes use of a ladder metaphor in which the top rung (rung 10) represents the individuals with highest status and the bottom rung (rung 1) represents the individuals with lowest status. The participants are asked to indicate on which of the 10 possible rungs they place themselves. Three categories of subjective social status were determined according to the respondents’ answers, i.e., low subjective social status (scores 1–4), middle subjective social status (scores 5–7) and high subjective social status (scores 8–10).

Social status in the adolescent sample was obtained using the German version of the Revised Family Affluence Scale (FAS III) (Torsheim et al., 2016). The scale consists of six items asking the participants about their family’s material assets, e.g., if they own a dishwasher or how often they have been on a holiday abroad in the past year. A total score is obtained by adding up answers across items. Cut-offs to determine categories were defined according to quintiles. Three categories were distinguished, e.g., low (bottom 20%, scores 0–5), middle (middle 60%, scores 6–9), and high (top 20%, scores 10–14) social status (Moor et al., 2024; Corell et al., 2021).

#### 2.3.2 SCOFF

The Sick, Control, One Stone, Fat, Food (SCOFF) questionnaire is a questionnaire initially developed to screen for anorexia nervosa and bulimia nervosa (Morgan et al., 1999). Here, the German translation was used (Hölling and Schlack, 2007). The questionnaire consists of five items conveyed as questions, each addressing one the core features of anorexia nervosa and bulimia nervosa, e.g., concerning recent weight loss and loss of control when eating. Participants are asked to answer each question with “yes” or “no” and a sum score is calculated across all items. If two or more questions are answered with “yes,” participants are considered likely to have an eating disorder. Across validation studies, the SCOFF questionnaire has yielded high sensitivity and specificity (Kutz et al., 2020; Botella et al., 2013). In addition, moderate to high test–retest reliability coefficients were obtained (Garcia et al., 2011; Garcia-Campayo et al., 2005; Leung et al., 2009).

#### 2.3.3 Body image

To assess the participants’ subjective body image, they were asked to indicate if they considered themselves to be exactly the right weight, a bit too thin, far too thin, a bit too fat or far too fat (Cohrdes et al., 2019). Three groups were determined: (1) individuals who consider themselves to have the right weight, (2) individuals who consider themselves to be a bit too thin or fat and (3) individuals who consider themselves to be far too thin or fat.

#### 2.3.4 HLS-EU-Q16

Health literacy was assessed using the German translation of the short version of the European Health Literacy Survey instrument (HLS-EU-Q16) (Jordan and Hoebel, 2015; Pelikan et al., 2014; Sørensen et al., 2013). The questionnaire consists of 16 items, answered on a 4-point Likert scale ranging from “very easy,” over “fairly easy,” “fairly difficult” to “very difficult.” Questions address subjective difficulty in accessing, understanding, appraising and applying information concerning healthcare, disease prevention and health promotion (Sørensen et al., 2013). To calculate an overall sum score, item responses were first dichotomized (e.g., 1 = “fairly easy” and “very easy,” 0 = “fairly difficult” and “very difficult”) and then added up across all dichotomized items. Levels of health literacy were determined according to the overall scores, i.e., inadequate or problematic health literacy for scores from 0 to 12 and adequate health literacy for scores from 13 to 16. The HLS-EU-Q16 questionnaire has yielded high internal consistency and test–retest reliability coefficients (Jordan and Hoebel, 2015; Eronen et al., 2019; Bergman et al., 2023).

### 2.4 Statistical analysis

The statistical analyses were conducted with the statistical software SPSS (version 29.0.2.0, IBM). Individuals with missing data for one construct or questionnaire item were excluded from all analyses concerning these constructs or questionnaires. To analyze whether the proportions of individuals identified as likely having an eating disorder by the SCOFF questionnaire were independent of the categorical sociodemographic and psychological factors studied here (i.e., gender, age, education, social status, body image, health literacy), χ^2^-tests of independence were calculated. Effect sizes for these tests are reported as Cramer’s *V.* For χ^2^-tests of contingency tables larger than 2 × 2, significant results were followed up with post-hoc Bonferroni-adjusted *z*-tests.

## 3 Results

### 3.1 Adults

#### 3.1.1 Sample characteristics adults

The adult sample consisted of *N* = 3,011 individuals. The sample characteristics. i.e., gender, age, level of education, social status, body image and health literacy categories, before (unweighted) and after (weighted) the weighting procedure can be found in Table 1.

**Table 1:** Sample characteristics of the weighted and unweighted sample of adults.

Variable	Unweighted sample, n (%)	Weighted sample, n (%)
Gender		
Male	1,483 (49.3%)	1,475 (49.0%)
Female	1,528 (50.7%)	1,536 (51.0%)
Age groups		
16–29 years	535 (17.8%)	531 (17.6%)
30–45 years	658 (21.9%)	721 (24.0%)
46–64 years	946 (31.4%)	976 (32.4%)
≥ 65 years	872 (29.0%)	783 (26.0%)
Level of education		
Low	768 (25.5%)	691 (23.0%)
Middle	1,110 (36.9%)	1,147 (38.1%)
High	1,130 (37.5%)	1,170 (38.8%)
Missing values	3 (0.1%)	3 (0.1%)
Social status		
Low	418 (13.9%)	426 (14.2%)
Middle	1992 (66.2%)	1988 (66.0%)
High	597 (19.8%)	593 (19.7%)
Missing values	4 (0.1%)	4 (0.1%)
Body image (Do you think you are…)		
… exactly the right weight?	886 (29.4%)	865 (28.7%)
…a bit too thin/fat?	1707 (56.7%)	1719 (57.1%)
…far too thin/fat?	417 (13.8%)	427 (14.2%)
Missing values	1 (0.0%)	1 (0.0%)
Health literacy		
Inadequate/problematic	1,192 (39.6%)	1,201 (39.9%)
Adequate	1790 (59.4%)	1784 (59.2%)
Missing values	29 (1.0%)	26 (0.9%)

#### 3.1.2 Associations with likely eating disorders in adults

In the weighted total samples of adults, 19.6% (*N* = 590) screened positive on the SCOFF suggesting an increased likelihood of having an eating disorder. 80.2% of participants (*N* = 2,414) screened negative and 0.2% (*N* = 7) had missing values in at least one SCOFF item impeding the calculation of the sum score.

An overview of the proportions of positive SCOFF screens for all sociodemographic and psychological categories in the adult sample can be found in Table 2.

**Table 2:** Proportions of positive and negative SCOFF screens in the adult sample for the gender, age, level of education, social status, body image and health literacy categories.

	Likely to have an eating disorder (%)	Not likely to have an eating disorder (%)
Gender		
Male	18.4%	81.6%
Female	20.8%	79.2%
Age groups		
16–29 years	22.7%	77.3%
30–45 years	22.2%	77.8%
46–64 years	19.7%	80.3%
≥ 65 years	15.2%	84.8%
Level of education		
Low	18.2%	81.8%
Middle	20.9%	79.1%
High	19.2%	80.8%
Social status		
Low	29.2%	70.8%
Middle	18.6%	81.4%
High	16.0%	84.0%
Body image (Do you think you are…)		
… exactly the right weight?	7.2%	92.8%
…a bit too thin/fat?	18.9%	81.1%
…far too thin/fat?	48.1%	51.9%
Health literacy		
Inadequate/problematic	27.1%	72.9%
Adequate	14.7%	85.3%

A positive SCOFF screen, defined as two or more “yes”-responses on the five SCOFF items, classifies individuals as likely to have an eating disorder. To confirm the presence of an eating disorder, positive screens need to be followed up with a more in-depth clinical assessment.

At the descriptive level, women were more likely to screen positive for eating disorders (20.8%) than men (18.4%), but this association was not significant, χ^2^ (1, *N* = 3,004) = 2.71, *p* = 0.100, *V* = 0.03. There was a significant association between age and the SCOFF categories, χ^2^ (3, *N* = 3,004) = 15.66, *p* = 0.001, *V* = 0.07. Descriptively, the proportions of positive SCOFF screenings decreased with increasing age (16–29 years: 22.7%; 30–45 years: 22.2%; 46–64 years: 19.7%; ≥ 65 years: 15.2%). Post-hoc tests revealed that the 16–29 years and 30–45 years groups did not differ significantly from each other and from the 46–64 years group, but from the ≥65 years group. There were no further significant differences between the age groups. The proportion of individuals scoring positive on the SCOFF did not differ by level of education, χ^2^ (2, *N* = 3,001) = 2.24, *p* = 0.327, *V* = 0.03, with similar rates in low (18.2%), middle (20.9%) and high (19.2%) educational levels. However, there was a significant association between subjective social status and fulfilling the SCOFF screening criterion, χ^2^ (2, *N* = 3,001) = 30.58, *p* < 0.001, *V* = 0.10. Post-hoc tests revealed that participants with lower subjective social status were more likely to screen positive for eating disorders than participants with middle and high subjective social status (low: 29.2%, middle: 18.6%, high: 16.0%), whereas the middle and high subjective social status groups did not significantly differ from each other.

Body image was significantly associated with fulfilling the SCOFF screening criterion, χ^2^ (2, *N* = 3,004) = 303.27, *p* < 0.001, *V* = 0.32. Participants who considered themselves to be far too thin or far too fat were more likely to screen positive for eating disorders (48.1%) than those who considered themselves to be a bit too thin or fat (18.9%) and those who believed to have exactly the right weight (7.2%). Post-hoc tests revealed that these differences were significant for all three groups.

There was a significant association between the SCOFF and health literacy categories, χ^2^ (1, *N* = 2,983) = 69.26, *p* < 0.001, *V* = 0.15, indicating lower proportions of positive SCOFF screenings in individuals with adequate (14.7%) vs. inadequate and problematic health literacy (27.1%).

### 3.2 Adolescents

#### 3.2.1 Sample characteristics adolescents

The adolescent sample consisted of *N* = 1,021 individuals. The sample characteristics. i.e., gender, age, level of education, social status, body image and health literacy categories, before (unweighted) and after (weighted) the weighting procedure can be found in Table 3.

**Table 3:** Sample characteristics of the weighted and unweighted sample of adolescents.

Variable	Unweighted sample, n (%)	Weighted sample, n (%)
Gender		
Male	514 (50.3%)	527 (51.6%)
Female	504 (49.4%)	492 (48.2%)
Diverse	3 (0.3%)	3 (0.3%)
Age groups		
12–13 years	340 (33.3%)	339 (33.2%)
14–15 years	346 (33.9%)	343 (33.6%)
16–17 years	335 (32.8%)	339 (33.2%)
Level of education		
Low	603 (59.1%)	603 (59.1%)
High	418 (40.9%)	418 (40.9%)
Social status		
Low	115 (11.3%)	114 (11.2%)
Middle	668 (65.4%)	666 (65.2%)
High	238 (23.3%)	241 (23.6%)
Body image (Do you think you are…)		
… exactly the right weight?	562 (55.0%)	565 (55.4%)
…a bit too thin/fat?	403 (39.5%)	400 (39.2%)
…far too thin/fat?	56 (5.5%)	56 (5.4%)
Health literacy		
Inadequate/problematic	548 (53.7%)	544 (53.3%)
Adequate	473 (46.3%)	477 (46.7%)

#### 3.2.2 Associations with likely eating disorders in adolescents

In the weighted total samples of adolescents, 18.9% (*N* = 193) screened positive on the SCOFF suggesting an increased likelihood of having an eating disorder. 81.1% of participants (*N* = 828) screened negative. There were no missing values.

An overview of the proportions of positive SCOFF screens for all sociodemographic and psychological categories in the adolescent sample can be found in Table 4.

**Table 4:** Proportions of positive and negative SCOFF screens in the adolescent sample for the gender, age, level of education, social status, body image and health literacy categories.

	Likely to have an eating disorder (%)	Not likely to have an eating disorder (%)
Gender		
Male	14.6%	85.4%
Female	23.4%	76.6%
Age groups		
12–13 years	13.9%	86.1%
14–15 years	18.0%	82.0%
16–17 years	24.8%	75.2%
Level of education		
Low	18.2%	81.8%
High	19.9%	80.1%
Social status		
Low	18.4%	81.6%
Middle	17.1%	82.9%
High	23.7%	76.3%
Body image (Do you think you are…)		
… exactly the right weight?	9.2%	90.8%
…a bit too thin/fat?	29.5%	70.5%
…far too thin/fat?	41.8%	58.2%
Health literacy		
Inadequate/problematic	24.6%	75.4%
Adequate	12.4%	87.6%

A positive SCOFF screen, defined as two or more “yes”-responses on the five SCOFF items, classifies individuals as likely to have an eating disorder. To confirm the presence of an eating disorder, positive screens need to be followed up with a more in-depth clinical assessment.

Girls were significantly more likely to screen positive for eating disorders (23.4%) than boys (14.6%), χ^2^ (1, *N* = 1,018) = 12.89, *p* < 0.001, *V* = 0.11. There was a significant association between age groups and the SCOFF categories, χ^2^ (2, *N* = 1,022) = 13.43, *p* = 0.001, *V* = 0.12. Descriptively, the proportions of positive SCOFF screenings increased with increasing age (12–13 years: 13.9%, 14–15 years: 18.0%, 16–17 years: 24.8%). Post-hoc tests revealed significant differences between the youngest and oldest groups, but not differences between the 14–15-year-olds and any other group. The proportion of adolescents scoring positive on the SCOFF did not differ by level of education, χ^2^ (1, *N* = 1,021) = 0.42, *p* = 0.517, *V* = 0.02, with similar rates in low (18.2%) and high (19.9%) educational levels. There was also no significant association between social status and fulfilling the SCOFF screening criterion, χ^2^ (2, *N* = 1,021) = 4.96, *p* = 0.084, *V* = 0.07. Descriptively, participants with lower and middle social status were less likely to screen positive for eating disorders than participants with high social status (low: 18.4%, middle: 17.1%, high: 23.7%).

Body image was significantly associated with fulfilling the SCOFF screening criterion, χ^2^ (2, *N* = 1,020) = 82.75, *p* < 0.001, *V* = 0.29. Participants who considered themselves to be a bit or far too thin or fat were significantly more likely to screen positive for eating disorders (29.5 and 41.8%, respectively) than individuals who believed to have exactly the right weight (9.2%). There was no significant difference between the individuals who considered themselves a bit vs. far too thin or fat.

There was a significant association between SCOFF and health literacy categories, χ^2^ (1, *N* = 1,021) = 24.93, *p* < 0.001, *V* = 0.16, indicating lower proportions of positive SCOFF screenings in adolescents with adequate (12.4%) vs. inadequate and problematic health literacy (24.6%).

## 4 Discussion

### 4.1 Principal findings

The present study investigated the suspected presence of eating disorders in two representative samples of the German-speaking population in Germany and its association with sociodemographic and psychological factors including body image and health literacy.

Overall, 19.6% of adults and 18.9% of adolescents screened positive for eating disorders. For adolescents, these proportions are largely in line with previous investigations in Germany (Herpertz-Dahlmann et al., 2008; Cohrdes et al., 2019). However, for adults, results are substantially higher than in a previous investigation (Richter et al., 2017). This discrepancy might reflect an increase in potential eating disorder prevalences in the last years, previously reported outside Germany (Romano et al., 2022) or it might be due to methodological differences between our own and the previous study (Richter et al., 2017). The same questionnaire might produce different results when used in an online compared to an offline sample, e.g., due to social desirability (Zhang et al., 2017; Richter et al., 2017).

Among adolescents, large and significant gender differences were delineated. Specifically, girls were more likely to screen positive for eating disorders than boys, which is consistent with previous meta-analytic findings from global data (López-Gil et al., 2023). In our sample, rates of positive screenings for eating disorders in boys were slightly lower than in the same meta-analysis (López-Gil et al., 2023) but broadly similar to previous investigations in German-speaking samples (Herpertz-Dahlmann et al., 2008; Cohrdes et al., 2019; Zeiler et al., 2016). In contrast, rates in girls were lower in our sample when compared to the meta-analysis and previous investigations in German-speaking samples (Herpertz-Dahlmann et al., 2008; Cohrdes et al., 2019; Zeiler et al., 2016), which might be cautiously interpreted as an overall negative trend over time, previously only observed in boys (Cohrdes et al., 2019). However, this can only be determined more in depth when screening questionnaires are followed up with detailed diagnostic interviews in positively screened individuals.

Surprisingly, there were no significant gender differences in the adult sample, which contrasts previous findings of increased prevalences of eating disorders in women vs. men (Solmi et al., 2014), but is consistent with previous reports of decreased gender differences in the SCOFF questionnaire with increasing age (Herpertz-Dahlmann et al., 2015; Richter et al., 2017).

In both samples, the likely presence of eating disorders was associated with age. In adolescents, an increase from the younger to the older age groups was observed, as in a previous meta-analysis (López-Gil et al., 2023) and other investigations in German speaking samples (e.g., Herpertz-Dahlmann et al., 2008). In the adult sample, the proportions of positive SCOFF screens decreased with increasing age, also in line with previous research (Solmi et al., 2014). Together, the results point to a peak of suspected eating disorder presence in late adolescence and early adulthood (Herpertz-Dahlmann, 2015). Prevention programs should therefore specifically target this age group.

Levels of education were not significantly associated with positive SCOFF screens in either sample. This finding is consistent with previous research with mixed or null results concerning the association of educational levels and eating disorders (Mitchison and Hay, 2014; Barakat et al., 2023; Solmi et al., 2014).

Social status was significantly associated with positive SCOFF screens only in adults, where higher proportions of suspected eating disorders were found in individuals with low compared to middle and high subjective social status. Descriptively, this pattern of results was reversed in adolescents (i.e., higher proportions with higher social status), but differences did not reach statistical significance. Importantly, social status was assessed differently in both samples, which is why results are not entirely comparable. In adults, social status was assessed with a subjective ranking, which might more closely mirror the perceived social structure of society. Conversely, the approach in adolescents might more specifically represent socioeconomic status as the items refer to material assets in the family. Overall, no clear associations of social status and eating disorders have been identified (Barakat et al., 2023).

As in previous studies (Cohrdes et al., 2019; Štefanová et al., 2020), subjective body image was significantly associated with suspected eating disorders in both groups. Critically, in both samples, proportions of positive eating disorder screens above 40% were found in those who considered themselves far too thin or far too fat. This is particularly worrying since shape or weight concerns are key features of both anorexia nervosa and bulimia nervosa (Treasure et al., 2020). However, no objective measure of weight or body mass index was obtained in this study, which is why subjective body image cannot be put into context of actual overweight or underweight. Cautiously, the results suggest that body image should be regarded as particularly important factors to address in future prevention programs. First approaches focusing on body image have been developed and evaluated but more high-quality research is needed to assess their effectiveness in reducing eating disorder symptoms or prevent onset of eating disorders (Yager and O'Dea, 2008; Le et al., 2017; Koreshe et al., 2023).

To our knowledge, this was the first study to link potential eating disorders with health literacy in large population-representative samples in Germany. In both our samples, higher proportions of suspected eating disorders were found in individuals with problematic or inadequate health literacy than in those with adequate health literacy. This finding is in line with previous research (Campanino et al., 2023; Boberová and Husárová, 2021). Although the cross-sectional design of our study precludes causal interpretation, our results suggest that health literacy may be an important factor to integrate into prevention strategies for eating disorders. First programs to enhance health literacy have been conceptualized and evaluated (Visscher et al., 2018; Walters et al., 2020). However, there is still a need for more targeted interventions, higher quality evaluations and a broader dissemination of positively-evaluated programs (Visscher et al., 2018; Walters et al., 2020).

### 4.2 Limitations

The results of this study have to be interpreted in the light of several limitations.

First, the cross-sectional design of the study does not allow causal interpretations of the association of the investigated constructs (Wang and Cheng, 2020). This means, for example, that we cannot unequivocally identify low health literacy as a risk factor for eating disorders as the direction of the association might be reversed.

Second, the adult sample is only representative of the German-speaking population with internet access in Germany. This means, that the results cannot be generalized to individuals without internet access or with limited knowledge of the German language.

Third, a recent meta-analysis points to reduced sensitivity of the SCOFF when applied in community samples compared to more homogeneous samples (such as young females) (Kutz et al., 2020), which has also been found in Germany (Richter et al., 2017).

Fourth, the SCOFF was not designed to address other—arguably even more prevalent—eating disorders such as binge eating disorders (Morgan et al., 1999; Kutz et al., 2020). A revision and extension of the questionnaire covering this topic might be necessary in the future.

Fifth, this study focused on potential eating disorders. However, some of the constructs reported here, such as negative body image, might not only be related to eating disorders but also to other psychiatric conditions such as mood disorders that are not the focus of the present work (Paans et al., 2018). Future studies should address potential associations between body image, different psychiatric conditions and eating behaviors, especially due to their high comorbidity (Preti et al., 2009).

Lastly, a further limitation concerning the SCOFF questionnaire is a potential inaccuracy in the German translation, which has been pointed out before (Zeiler et al., 2016) and might inflate the number of positive SCOFF screenings. The English verb “dominates” in item 5 was translated to “beeinflusst sehr” which can be back-translated to “influences a lot.” In our sample, as in other German samples (Zeiler et al., 2016; Herpertz-Dahlmann et al., 2015), the rate of positive responses to this question is higher than in non-German speaking samples (Watson et al., 2015; e.g., Peat et al., 2015). Specifically, in the present study, 40.7% of adults and 26.0% of adolescents gave an affirmative response to this item. However, overall rates of positive SCOFF screenings in our samples are not higher than in a recent meta-analysis (López-Gil et al., 2023). This can be interpreted in two ways: either the inaccurate translation does not negatively impact the diagnostic accuracy of the SCOFF or prevalences of likely eating disorders are in fact lower in Germany but inflated by the disproportionate number of affirmations of item 5. Taken together, we propose a re-evaluation of the German version of the SCOFF to make it more comparable to other language versions.

### 4.3 Summary and conclusion

This was the first study to comprehensively investigate the associations of the suspected presence of eating disorders, particularly anorexia nervosa and bulimia nervosa, with several sociodemographic variables as well as body image and health literacy in two large and population-representative samples of German-speaking adolescents and adults. Suspected eating disorders were more likely in female than male adolescents but were not related to gender in adults. Highest rates of suspected eating disorders were found in late adolescence and early adulthood. While levels of education were unrelated to suspected eating disorders, low subjective social status was associated with higher rates of suspected eating disorders in adults but not adolescents. Individuals with low levels of health literacy and negative body image had higher levels of suspected eating disorders than individuals with adequate health literacy and more positive body image. The overall high rates of suspected eating disorders in both samples highlight the need for more preventive programs to lower the overall burden of the disorders. These could be targeted particularly at female individuals in late adolescence and early adulthood and should address aspects of body image and health literacy.

## Data Availability

The datasets generated and analyzed during this study are the property of the independent, nonprofit foundation Stiftung Gesundheitswissen and are available on reasonable request. Requests to access the datasets should be directed to lars.koenig@stiftung-gesundheitswissen.de. This study was part of a larger study, and therefore the raw data set contains further variables that have not been described because they exceed the scope of this study.
